# Learning curves in minimally invasive pancreatic surgery: a systematic review

**DOI:** 10.1007/s00423-022-02470-3

**Published:** 2022-03-12

**Authors:** Gayle Fung, Menazir Sha, Basir Kunduzi, Farid Froghi, Saad Rehman, Saied Froghi

**Affiliations:** 1 Barts and The London School of Medicine and Dentistry, Queen Mary University of London, London, UK; 2 Medical School, University College London, London, UK; 3 Guy’s Hospital, Renal Transplant Unit, London, UK; 4 Department of HPB & Liver Transplantation, Royal Free Hospital, Pond St, Hampstead, NW3 2QG London UK; 5 Division of Surgery & Interventional Sciences, Royal Free Campus, University College London, Hampstead, , London UK; 6 Upper GI & Bariatric Unit, Royal Shrewsbury Hospital, Shrewsbury, UK

**Keywords:** Learning curve, Minimally invasive surgery, Pancreatic surgery, Training, Laparoscopic, Robotic

## Abstract

**Background:**

The learning curve of new surgical procedures has implications for the education, evaluation and subsequent adoption. There is currently no standardised surgical training for those willing to make their first attempts at minimally invasive pancreatic surgery. This study aims to ascertain the learning curve in minimally invasive pancreatic surgery.

**Methods:**

A systematic search of PubMed, Embase and Web of Science was performed up to March 2021. Studies investigating the number of cases needed to achieve author-declared competency in minimally invasive pancreatic surgery were included.

**Results:**

In total, 31 original studies fulfilled the inclusion criteria with 2682 patient outcomes being analysed. From these studies, the median learning curve for distal pancreatectomy was reported to have been achieved in 17 cases (10–30) and 23.5 cases (7–40) for laparoscopic and robotic approach respectively. The median learning curve for pancreaticoduodenectomy was reported to have been achieved at 30 cases (4–60) and 36.5 cases (20–80) for a laparoscopic and robotic approach respectively. Mean operative times and estimated blood loss improved in all four surgical procedural groups. Heterogeneity was demonstrated when factoring in the level of surgeon’s experience and patient’s demographic.

**Conclusions:**

There is currently no gold standard in the evaluation of a learning curve. As a result, derivations are difficult to utilise clinically. Existing literature can serve as a guide for current trainees. More work needs to be done to standardise learning curve assessment in a patient-centred manner.

## Introduction

One of the earliest reported incidents of minimally invasive surgery (MIS) of the abdomen dates back to the 1910s at John Hopkins for an ‘organoscopy’ to stage pancreatic cancer [1]. With improvements in fibre-optics post-world war 2 and the ingenious work of several pioneers since laparoscopic surgery continued to surge in the 1980s—with the first laparoscopic appendectomy and cholecystectomy. Minimally invasive techniques bear the possibility of superior surgical and post-operative outcomes, enhanced dexterity and improved cosmesis [2]. The current decade has witnessed further strides with robotic surgery which boasts the ability to overcome the shortcomings of laparoscopy [3]. As with the adoption of any new surgical approach, there exists an initial training period with performance outcomes that improve with experience. The surgical learning curve has previously been defined as ‘the time taken and/or the number of procedures an average surgeon needs to be able to perform a procedure independently with a reasonable outcome’ [4]. It is traditionally described to have three phases: a starting point; a slope and a plateau. A surgical learning curve is achieved when a stage (case number) is reached where outcomes (operative time, operative blood loss, etc.) demonstrate a maximal rate of improvement from the overall performance.

The learning curve is influenced by a variety of factors, including the nature of the procedure being performed, surgical workload, choice of surgical instruments and technologies, training programme and the innate ability of the individual surgeon. Patient factors such as case-mix and anatomy can also influence the learning curve and should not be overlooked [4]. In addition to the manual skills of operating, it is known that non-technical skills such as informed decision-making and the ability to make pressured intraoperative decisions also contribute to the learning curve [5]. As such, efforts into understanding the learning curve of a particular surgical procedure can highlight the significance of improvements in a multifactorial performance environment. Pancreatic surgery encompasses technically demanding procedures such as distal pancreatectomy (DP) and pancreaticoduodenectomies (PD). Learning curve outcomes can help set the framework for establishing surgical competencies when developing specific training curriculums for minimally invasive pancreatic surgeries on an individual level.

Looking at an institutional level, minimally invasive pancreatic procedures have mainly gained traction and success in a select number of high-volume pancreatic centres, where surgeons are well versed with minimally invasive procedures [6]. With a thorough assessment of the nature of the learning curve and the number of cases required for the achievement, other centres will be better informed to project outcomes in the early stages of adoption. This can facilitate the implementation of minimally invasive pancreatic surgery techniques on a wider scale.

Pancreatic surgery also has the added challenge of operating on an elderly demographic with multiple comorbidities. Efforts to delineate the learning curve would not only be useful to improve the standardisation of surgical training but also to help reduce patients being disadvantaged to suboptimal outcomes of the initial learning curve period.

This review aims to thoroughly assess the metrics surrounding surgical competency in existing literature, the overall cases required for competency in a new technique to be achieved and ultimately serve in the subsequent evaluation of minimally invasive laparoscopic techniques in pancreatic surgery.

## Materials and methods

### Study selection

This study was carried out per guidelines from the Preferred Reporting Items for Systematic reviews and Meta-Analyses (PRISMA) statement [7].

An electronic search of PubMed, Embase and Web of Science databases was carried out in June 2020 using the following search terms: ‘pancreas’ OR ‘pancreatic’ AND ‘learning curve’ AND ‘minimally invasive’ OR ‘robotic’ OR ‘laparoscopic’. Results were limited to English language articles published between January 2000 and March 2021. Empirical studies concerning the learning curve in minimally invasive, whether laparoscopic, robotic or hybrid in nature, pancreatic surgery were included. Systematic review articles, animal/model studies, letters and comments were excluded.

Two reviewers (G.F. & M.M.) independently identified potentially relevant articles. Full texts were obtained for relevant abstracts and each screened for inclusion. Conflict between reviewers was subsequently discussed, such that agreement was > 0.85 (Cohen coefficient). All original papers presenting outcome measures in the context of a learning curve were included. Conference abstracts concluding on a number for the learning curve were included too. The bibliographies of full articles were manually searched for any further relevant articles.

#### CUSUM calculation

Nineteen studies used the cumulative sum method (CUSUM) [8–26] for graphically assessing trends in data. This method illustrates the learning curve by calculating and plotting the difference between the raw data point and the mean value of all the previous data points. Patient cases are ordered chronologically from earliest to latest date of surgery. Therefore, the CUSUM-operative time (CUSUM-OT) for the first patient is the difference between the OT for the first patient and the mean OT for all patients. Similarly, the CUSUM-OT of the second patient is the CUSUM-OT of the previous operation plus the difference between the OT of the second patient and the mean OT for all patients.

The learning curve is then graphically represented by plotting CUSUM over the number of cases. The flexion point where the slope begins to descend is inferred as the number of cases required to achieve the learning curve in these studies.

Furthermore, four studies [16, 17, 21, 27] also used the risk-adjusted CUSUM (RA-CUSUM) method which accounts for confounding factors such as case mix by allowing comparison of actual risk with expected risk.

### Data collection and statistical analysis

An electronic data collection proforma (Microsoft Excel 2007, Redmond, WA, USA) was used to extract and store data from included articles. This constituted the type of procedure, number of surgeons/patients, previous surgical experience, statistical analyses methods, outcome measures used to measure learning curve and learning curve itself. Traditionally, the learning curve can be assessed using surgical and patient outcomes. We screened for such parameters that were reported. Patient demographics, operative and post-operative outcomes were also documented. Disagreements in the assessment and data extraction were resolved by consensus (G.F. & M.M.).

Due to the heterogeneous study designs and lack of comparative variables, direct comparisons or meta-analysis of data was not feasible. Some studies omitted aspects of patient demographics and the experience of participating surgeons. However, if identical tools or outcome measures were used in different studies, the results for the different items of the framework used were summarised. Where possible, the collated data were divided into ‘early experience’ and ‘late experience’ for statistical analysis. Early experience was defined as those on the initial phase of their learning curve and the late experience was defined as those subjects who had reached the plateau phase of their learning curve. Where reported, operative and estimated blood loss differences between early and late experience groups were analysed using an independent *t*-test (IBM Corp. Released 2017. IBM SPSS Statistics for Macintosh, Version 25.0. Armonk, NY: IBM Corp).

IRB was not needed for this study.

### Quality assessment:

A qualitative assessment of the studies was carried out (GRADEpro Guideline Development Tool, McMaster University, 2020, developed by Evidence Prime, Inc.) assessing their: study design, risk of bias, inconsistency, indirectness and imprecision. The grading system then scores the study from very low (⨁◯◯◯) to high (⨁⨁⨁⨁).

## Results

### Study selection

**Fig. 1:**
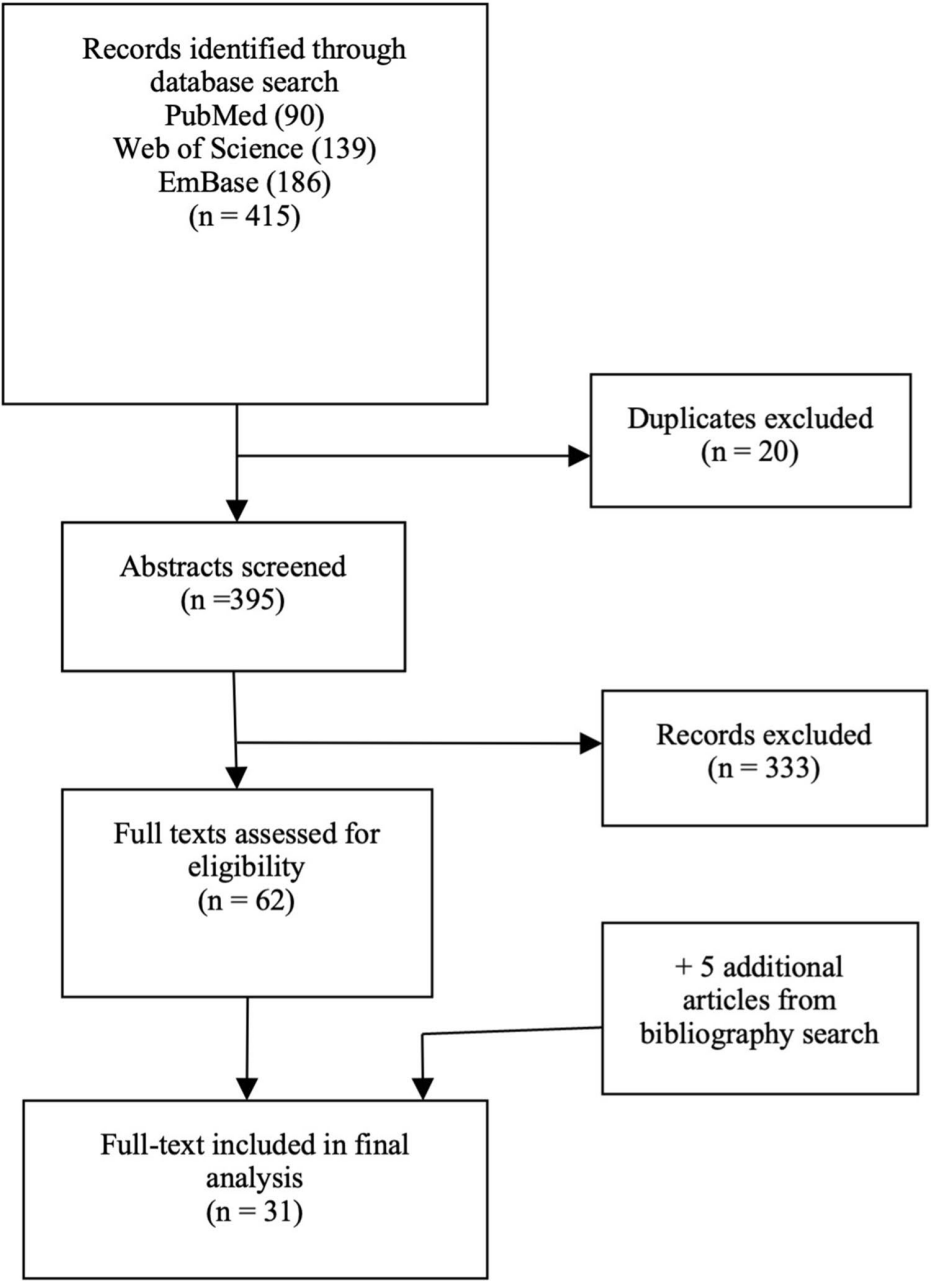
PRISMA flow diagram of study selection

A total of 415 potential studies were identified by the systematic search. Twenty duplicates were removed and 395 abstracts were reviewed. Of these, 62 abstracts were deemed relevant and full texts were obtained if possible. From these, 26 studies met the inclusion criteria. A screen of the bibliographies disclosed five additional relevant studies leading to a total of 31 studies. A flowchart of the selection process is demonstrated in Fig. 1.

### Study characteristics

Thirteen studies were performed retrospectively [10, 11, 16, 17, 19, 20, 24, 27–32], 9 studies [8, 12–14, 22, 23, 33–35] were retrospective evaluations of prospectively collected data and the remaining 9 studies were prospective [9, 15, 21, 25, 26, 36–38]. Fifteen of the studies (50%) analysed the learning curve corresponding to a single surgeon [8, 11, 13, 16, 17, 21, 22, 24, 26, 27, 29, 33, 35, 38, 39]. Where indicated, group sizes ranged from 2 to 15 surgeons [12, 14, 15, 19, 23, 25, 31, 36]. Five studies presented outcomes pertaining to the learning curve of a team without specifying the number [9, 10, 20, 30, 37], and two studies did not explicitly say how many surgeons were involved in the study [28, 34]. The median patient sample size was 70 (range 11–211) and 35.5% did not indicate the prior surgical experience of the surgeon(s). The procedures described can be divided into distal pancreatectomy and pancreaticoduodenectomy, both of which can be subdivided into laparoscopic and robotic. Study characteristics can be seen in Tables 1 and 2.

**Table 1:** Study characteristics for distal pancreatomy procedures

Study name	GRADEpro certainty	Procedure type	Study type	No. of cases	Matching	Mean age, years	Study quality
Barrie et al. 2015 [8]	⨁⨁⨁◯ MODERATE	L	RP	25	1,4,5,6	54	******
Belgaumkar et al. 2016* [26]	⨁⨁⨁◯ MODERATE	L	P	94			****
Benizri et al. 2014 [12]	⨁⨁◯◯ LOW	R	RP	11	1,2,3,4,5,6,7	51.6	*****
Braga et al. 2012 [9]	⨁⨁⨁⨁ HIGH	L	P	30	1,2,3,4,6,8	55.5	*******
De Rooij et al. 2017 [33]	⨁⨁⨁◯ MODERATE	L	RP	111	1,3,4,5,6,	62	*****
Hua et al. 2017 [34]	⨁⨁⨁◯ MODERATE	L	RP	211	1,2,3,4,5,6,7,		******
Kim et al. 2018 [10, 17]	⨁⨁⨁⨁ HIGH	L	R	83	1,2,4,5,6,7	54.8	*******
Napoli et al. 2015 [13]	⨁⨁⨁⨁ HIGH	R	RP	55	1,2,3,4,6,7,	56.6	******
Ricci et al. 2014	⨁⨁⨁◯ MODERATE	L	R	32	1,2,3,4,5,6,7	57	*******
Shakir et al. 2015 [14]	⨁⨁⨁⨁ HIGH	R	RP	100	1,2,3,4,5,6,7,8	60.13	*****
Shyr et al. 2018 [15]	⨁⨁⨁⨁ HIGH	R	P	70	1,2,3,6	55	*****

Matching: 1, age; 2, BMI; 3, ASA; 4, gender; 5, tumour size; 6, pathology; 7, prior surgery; 8, Pre-op albumin. Study type: *R*, retrospective; *P*, prospective; *RP*, retrospective evaluation of prospectively collected data. Procedure type: *L*, laparoscopic; *R*, robotic. Studies marked * are conference abstracts

**Table 2:** Study characteristics for pancreaticoduodenectomy procedures; studies marked * are conference abstracts

Study name	GRADE pro quality	Procedure type	Study type	No. of cases	Matching	Mean age, years	Study quality
Boone et al. 2015 [23]	⨁⨁⨁⨁ HIGH	R	RP	200	1,2,4,5,6,7	67	******
Chen et al. 2014	⨁⨁⨁◯ MODERATE	R	P	60	1,2,3,4,5,6,7,8	53.6	****
Corcione et al. 2012	⨁⨁⨁◯ MODERATE	L	R	22	1,4,6	62	*****
Khatkov et al. 2013* [37]	⨁⨁⨁⨁ HIGH	L	P	43			****
Kim et al. 2012	⨁⨁⨁⨁ HIGH	L	R	100	1,2,4,6	50	*****
Kim et al. 2017 [38]	⨁⨁⨁◯ MODERATE	L	P	16	1,2,4,6	63.1	*****
Kim et al. 2018 [17]	⨁⨁⨁⨁ HIGH	L	R	90	6		******
Kuroki et al. 2014* [29]	⨁⨁⨁◯ MODERATE	L	R	30			***
Lu et al. 2016 [35]	⨁⨁⨁◯ MODERATE	L	RP	120	1,2,3,4,5,6,7,	59.7	*******
Nagakawa et al. 2018 [19]	⨁⨁⨁⨁ HIGH	L	R	150	1,2,4,5,6	69	*******
Napoli et al. 2016* [22]	⨁⨁⨁◯	R	RP	70			****

Matching: 1, age; 2, BMI; 3, ASA; 4, gender; 5, tumour size; 6, pathology; 7, prior surgery; 8, Pre-op albumin. Study type: *R*, retrospective; *P*, prospective; *RP*, retrospective evaluation of prospectively collected data. Procedure type: *L*, laparoscopic; *R*, robotic. Studies marked * are conference abstracts

### Patient demographics

Of the 2682 patients included in the studies, the most common indication for surgery was reported as adenocarcinoma, followed by peri/ampullary cancer and then intraductal papillary mucinous neoplasm. All other indications can be seen in Table 3. Where reported, the mean tumour size was 27.6 mm and the mean BMI was 24.3 kg/m^2^.

**Table 3:** Surgical indications where specified, total: 1176

Indication	No. of cases
*Adenocarcinoma*	423
*Peri/ampullary cancer*	315
*Intraductal papillary mucinous neoplasm*	207
*Endocrine/neuroendocrine tumour*	195
*Common bile duct cancer*	165
*Chronic pancreatitis*	137
*Serous cystic neoplasm*	62
*Solid pseudopapillary tumour*	56
*Mucinous cystic neoplasm*	53
*Metastasis*	26
*Ampullary adenoma*	12
*Autoimmune pancreatitis or cholangitis*	10
*Pseudocyst*	9
*Ampulla of Vater adenoma*	5
*Duodenal cancer*	5
*Mucinous cystic neoplasm or serous cystic neoplasm*	3
*Ganglioneuroma*	1
*Benign cyst*	1

### Assessment of the learning curve

Twenty-eight out of 31 studies derived their learning curve from based on improvements in operative time (surgical outcome). The second most commonly used surgical process outcome was estimated blood loss used by 14 studies [9, 10, 14–17, 19, 21, 23, 27, 30, 35, 36]. Patient outcomes were less used to generate the learning curve. These included conversion rates, fistula rates, length of hospital stay, post-operative morbidity, reoperation rates and mortality.

#### Laparoscopic distal pancreatectomy (LDP)

There were seven studies in total describing the learning curve of laparoscopic distal pancreatectomy (LDP) (Table 4). Four studies [8, 11, 26, 33] were based on the experience of a single surgeon, two studies were based on the experiences of a team [9, 10] and one group [34] did not specify how many surgeons were involved. A range of intraoperative and post-operative outcomes were used to define learning curve including operative time, Clavien-Dindo complications and conversion rate. The learning curve reported for this procedure ranges between 10 and 30 cases.

**Table 4:** Studies describing LDP learning curve

First author	No. of patients	No. of surgeons	Previous experience of surgeons	Outcome measures	Statistical analysis	Learning curve and results
Barrie and Ammori 2015 [8]	25	1	Extensive experience in complex laparoscopic operations	Operative time	Split group, CUSUM	10 for LDP + splenectomy, 11 for LDP + splenic preservation Mean OpTime with splenic preservation: 220 min and without 260 min (not statistically significant)
Braga et al. 2012 [9]	30	Team	Large experience in open pancreatic surgery	Operative time, conversion rate, operative blood loss	Split group, Fisher’s exact test, Student’s *t* test, CUSUM	**10** OT: 1–10 = 254 min, 11–20 = 206 min, 21–30 = 183 min EBL: 1–10 = 450 ml, 11–20 = 245 ml, 21–30 = 300 ml
Belgaumkar et al. 2016* [26]	94	1	-	No. of Clavien-Dindo complications	CUSUM, regression analysis	**30** Mean OpTime 210 min
de Rooij et al. 2017 [33]	111	1	Experience in open pancreatic surgery (~ 100 procedures); experience in laparoscopic GI surgery, experience in laparoscopic hepatectomy (~ 20 procedures)	ISGPF grade B/C pancreatic fistulas, Clavien-Dindo III or higher complications, length of hospital stay	Split group, chi-square test, independent samples *t*-test, Mann–Whitney *U* test	**30** OT remained stable throughout: 200 min Blood loss remained stable throughout: 200 ml
Hua et al. 2017 [34]	211	-	-	Conversion rate No time or blood loss mentioned to determine LC	Two-sample *t*-test, Mann–Whitney *U* test, chi-square test, Fisher exact test, multivariable logistic regression model, univariate analysis	15
Kim et al. 2018a [10]	65	Team	No experience	Operative time, estimated blood loss,	Chi-square test, Fisher’s exact test, independent two sample *t*-test, Mann–Whitney *U* test, CUSUM	16
Ricci et al. 2015 [11]	32	1	> 50 open pancreaticoduodenectomies/distal pancreatectomies	Operative time	ANOVA, linear regression analysis, Fisher’s exact test, Student’s *t*-test, CUSUM	17

Barrie et al. [8] investigated the learning curve in an ‘expert laparoscopic surgeon’. CUSUM analysis of operative time over 25 cases showed that the learning curve was overcome in 10 cases for LDP with splenectomy, whilst for LDP with splenic preservation, 11 cases are required. However, CUSUM analysis of blood loss showed a learning curve of around six cases. They also attempted split group analysis of the first and second group of consecutive patients but found no statistical difference between the two groups—citing small sample size and heterogeneity as a possible reason. Braga et al. [9] showed that in a surgical team experienced in open pancreatic surgery and other laparoscopic GI procedures, the learning curve was overcome after the first 10 cases indicated by a drop in mean operative time from 254 min in the first ten patients to 206 min in the next ten patients (*p* = 0.09 vs. first). The conversion rate to open surgery also dropped significantly.

Belgaumkar et al. [26] reported the outcomes of 94 LDP cases performed by a single surgeon. They were unable to show significant changes in either mean operative time improvements or operative blood loss—despite the increasing complexity of the case. Instead, they relied on analysing the frequency of complication rates (Clavien-Dindo type III + , readmissions and post-operative pancreatic fistula) to interpret the learning curve. The study reports that the number of Clavien-Dindo type III + complications peaked at 30 cases and significantly decreased subsequently. In the first 30 cases, there were eight Clavien-Dindo type III + complications compared to three occurring in the latter 64 patients. They thus inferred that this signified the end of the learning curve.

De Rooij et al. [33] published the first single-surgeon study in describing outcomes for the learning curve of more than 100 LDPs. They demonstrated that in a surgeon experienced in open pancreatic procedures and laparoscopic GI procedures, the learning curve was achieved by 30 cases. This was indicated by a significant decrease in ISGPF grade B/C pancreatic fistulas, Clavien-Dindo III + complications and length of hospital stay. Similar to Belgaumkar et al. [26], they were unable to find any improvements in intraoperative outcomes such as operative time or blood loss.

A retrospective study of over 211 LDP cases by Hua et al. [34] reported that the learning curve was achieved after 15 cases. They analysed various factors associated with the risk of conversion from laparoscopic to open distal pancreatectomies such as malignant disease, multiorgan resection and intraoperative factors (excessive intraabdominal and retroperitoneal fat). It was also highlighted that higher conversion rates were observed amongst surgeons who had lesser than 15 cases of experience.

Kim et al. [10] looked specifically at splenic vessel preserving LDP in a group of surgeons with no experience in laparoscopic pancreatic surgery. Using the CUSUM methods of analysis of 65 cases, they demonstrated that the rate of splenic vessel preserving LDP peaked at 16 cases—implying the end of the learning curve. They also found no significant points when looking at operative time or blood loss.

In a study by Ricci et al. [11] of 32 LDP performed by a single experienced surgeon, the learning curve was reported to have been achieved after 17 cases—determined by cumulative operative time. However, this was not concordant with the conversion and reoperation rates, overall post-operative morbidity and mortality rates and length of stay.

#### Robotic distal pancreatectomy (RDP)

There were four papers in total describing the learning curve for robotic distal pancreatectomies (RDP) (Table 5). The learning curve for this procedure ranged from 7 to 40 cases, and number of surgeons participating ranged from 1 to 3. All four studies used operative time as a parameter to represent the learning curve. It should be noted that only Benizri et al.’s [12] study involved surgeons trained specifically in robotic surgery. Their reported learning curve of 7 cases is the shortest compared to the other studies where the surgeons had no substantial prior robotic experience.

**Table 5:** Studies describing RDP learning curve

Study	No. of patients	No. of surgeons	Previous experience of surgeons	Outcome measures	Statistical analysis
Benizri et al. 2013 [12]	11	2	Board-certified with specific training in robotic procedures	Operative time, conversion, post-operative morbidity, reoperation rates	Mann–Whitney *U* test, linear estimation regression analysis, Fisher’s exact test, CUSUM analysis
Napoli et al. 2015 [13]	55	1	> 700 open or laparoscopic pancreatic resections	Operative time	Chi-square test, Fisher exact test, CUSUM analysis
Shakir et al. 2015 [14]	100	3	Extensive prior experience with LDP, but no substantial prior robotic experience	Operative time	Student’s *t*-test, ANOVA, Wilcoxon rank-sum test, Kruskal–Wallis test, Fisher’s exact test, CUSUM analysis
Shyr et al. 2018 [15]	70	2	No prior experience in robotic surgery, > 500 cases of open PD	Console time	2- tailed Student’s *t*-test, Pearson’s chi-square test, Fisher’s exact test, CUSUM analysis

Napoli et al. studied the learning curve in a single surgeon with previous experience in more than 700 open/laparoscopic pancreatic resections [13]. In this study of 55 cases, they demonstrated the completion of the learning curve after 10 cases in terms of operative time improvements using the CUSUM method. Other standard parameters such as conversion rates and need for blood transfusion were uniformly favourable throughout the 55 cases and thus unable to be delineated to mark a learning curve significantly.

In a study involving 3 surgeons and 100 patients, Shakir et al. [14] reported the completion of the learning curve at 40 cases. They further delineated the curve into three distinct phases. Phase 1 consisted of the first 20 cases where operative time stabilises. Although the next phase (cases 20–40) started to represent a reduction in operative time, this only became significant in Phase 3 (cases 40–100). They also observed marked reductions in the 90-day readmission rate, grade B/C fistulas and Clavien-Dindo III/IV complications after completion of the learning curve.

Shyr et al. reported the completion of the learning curve after 37 cases in a team of 2 surgeons involving 70 cases [15].

#### Laparoscopic pancreaticoduodenectomy (LPD)

There were 14 studies in total describing the learning curve in LPD (Table 6). Eight studies [16–18, 21, 29, 35, 38, 39] were single-surgeon based, three were based on the experience of a team, one described the experience of three surgeons, one was based on the experience of two surgeons and the remaining studies did not document how many surgeons were involved. All learning curves were derived from the analysis of operative time as a minimum parameter. The learning curve for this procedure ranged between 4 and 60 cases.

**Table 6:** Studies describing LPD learning curve

Study	No. of patients	No. of surgeons	Previous experience of surgeons	Outcome measures	Statistical analysis	Learning curve
Corcione et al. 2013 [28]	22	-	-	Operative time	Split group	11
Choi et al. 2020 [16]	171	1	-	Operative time, blood loss, length of stay, conversion rate, complications	CUSUM, RA-CUSUM, Student’s *t* tests ANOVA, chi-square test, Fisher’s exact test	40
Khatkov et al. 2013 [37]	43	Team	-	Operative time, intraopearative blood loss, length of post-operative morbidity	-	25
Kim et al. 2013 [39]	100	1	-	Operative time	Split group, Student’s *t*-test, chi-square test	33
Kim et al. 2017 [38]	16	1	-	Operative time	Student’s *t*-test, chi-square test	6
Kim et al. 2018 [17]	90	1	-	Operative time, blood loss, length of stay, surgical indication, complications	CUSUM, RA-CUSUM	10
Kim et al. 2020 [18]	119	1	252 open pancreatoduodenectomy (OPD) over 9 years	Operative time, blood loss, length of stay, conversion ate, complications	CUSUM, RA-CUSUM, Student’s *t* tests ANOVA, chi-square test, Fisher’s exact test	47
Kuroki et al. 2014 [29]	30	1	Experience in open PD and LPD	Operative time, blood loss	-	10
Lu et al. 2016 [35]	120	1	Prior experience with OPD and laparoscopic procedure such as radical gastrectomy, distal or central pancreatectomy	Operative time	Split group, ANOVA, Kruskal–Wallis test, Scheffe test	30–60
Nagakawa et al. 2018 [19]	150	3	Surgeon A: 18 years of surgical experience, > 500 laparoscopic procedures	Operative time	CUSUM, Mann–Whitney *U* test, Fisher’s exact test, multivariate analysis	30
			Surgeon B: 17 years of surgical experience, > 500 laparoscopic procedures			
			Surgeon C: 19 years of surgical experience, > 900 laparoscopic procedures			
Speicher et al. 2014 [30]	56	5	All procedures were performed by surgical teams consisting of a fellowship-trained minimally invasive surgeon and one of four surgical oncologists experienced in open pancreatic surgery	Operative time, blood loss	Split group, ANOVA, chi-square, Fisher’s exact test, Kruskal–Wallis test	50
Tyutyunnik et al. 2016 [20]	100	Team	-	Operative time	CUSUM	48
Wang et al. 2016 [21]	63	1	Experience of over 30 open pancreaticoduodenectomy procedures	Operative time	CUSUM, RA-CUSUM, ANOVA, chi-square test, Fisher’s exact test, Kruskal–Wallis test, multivariate logistic regression	40
			300 laparoscopic splenectomy procedures			
			4 laparoscopic spleen-preserving distal pancreatectomy operations			
			17 consecutive laparoscopic cholecystectomy and splenectomy procedures			
Yeo et al. 2017 [31]	20	2	Laparoscopically trained hepatobiliary and pancreatic surgeon	Operative time, blood loss	-	4

Studies marked * are conference abstracts

Corcione et al. [28] demonstrated a reduction in operative time in the last 11 procedures in a study of 22 patients. The first 11 procedures were between 480 and 570 min, whereas the last 11 procedures ranged between 327 and 480 min. Khatkov et al. [37] investigated the learning curve by the same surgical team over 5 years and demonstrated significantly better outcomes (operative time, intraoperative blood loss and level of post-op morbidity) after 25 procedures.

Kim et al. [39] determined a learning curve of 33 cases by looking at the operative time, complication rates and mean hospital stay.

Kim et al. [38] looked at the learning curve in a laparoscopic and robotic hybrid pancreaticoduodenectomy by measuring the actual operation time of a single surgeon. The operation time was defined as the sum of the resection time and anastomosis time. In this hybrid procedure, resection was performed by laparoscopy and anastomosis (pancreaticojejunostomy and hepaticojejunostomy) was performed with robot. In their experience, actual operation time became consistent after the 6th case marking the end of the learning curve. The study did not disclose the prior experience the operative surgeon might have had to achieve this. The study cited the ergonomic advantages of robotic surgery and virtual simulations that aided the anastomosis.

Kim et al. [17] found four phases to a learning curve in a single surgeon. Phase I was the initial learning period (cases 1–10), phase II was described as the technical stabilising period (cases 11–37), phase III was the second learning period (cases 38–70) and phase IV represented the secondary stabilising period (cases 71–90).

Kuroki et al. [29] examined a single surgeon’s learning curve by analysing operative time and blood loss and demonstrated a learning curve plateau after 10 cases. Lu et al. [35] found there was a clear reduction in operative time for laparoscopic pancreaticojejunostomy (LPJ) after the first 30 cases and for laparoscopic choledochojejunostomy (LCJ) after the first 60 cases. This was shown in a single surgeon with previous experience in performing various laparoscopic GI procedures. They stated the reason that they could not conclude a definite number for the learning curve was due to varied indications and specificity of previous experience. Nagakawa et al. [19] analysed the operative time in 150 consecutive cases of 3 surgeons during their first 50 cases and demonstrated that 30 cases are required to overcome the learning curve. Additionally, the learning curve can be divided into three phases. Case 1–20 represents the initial phase, cases 21–30 represent the plateau phase and cases 31–50 represent the stable phase.

Speicher et al. [30] carried out a study in advanced laparoscopic trained surgeons and advanced oncologic trained surgeons and demonstrated a significant decrease in operative time following the first 10 patients.

Tyutyunnik et al. [20] studied a single surgical team retrospectively and demonstrated a learning curve of 48 cases by applying a RA-CUSUM model to operative time. Wang et al. [21] reports that 40 cases are required to achieve technical competence in a surgeon with extensive open pancreaticoduodenectomy experience and some laparoscopic experience. Their learning curve based on operative time could further be divided into three phases. Phase 1 (cases 1–11) representing the initial learning curve, phase 2 (cases 12–38) representing increased competence and phase 3 (cases 39–57) which represented mastery of the procedure. Fewere surgical complications were seen at the 38th case and considered the point at which the learning curve was achieved.

Yeo et al. [31] evaluated the learning curve of two laparoscopically trained hepatobiliary and pancreatic consultants and saw a significant decrease in median operative time and median blood loss after the 4th case.

Two recent studies published in 2020 by Choi et al. [16] and Kim et al. [18] aimed to investigate the learning curve of LPD procedures over 171 and 119 cases respectively. Both these studies measured only a single surgeon’s performance. Measuring outcomes such as OT, EBL and failure rates, improvements were reached after 40 cases in Choi et al.’s study and 47 cases in Kim et al.’s study.

#### Robotic pancreaticoduodenectomy (RPD)

Six studies investigated the learning curve for RPD (Table 7). The number of participating surgeons ranged from 1 to 15 and the outcomes used to measure the learning curve included operative time, blood loss, readmission rate, surgical indication, morbidity and fistula rate. The learning curve for RPD ranged between 20 and 80.

**Table 7:** Studies describing RPD learning curve

Study	No. of patients	No. of surgeons	Previous experience of surgeons	Outcome measures	Statistical analysis	Learning curve
Boone et al. 2015 [23]	200	4	-	Operative time	Analyses of variance, 2-tailed unpaired *t* test, Kruskal–Wallis, Wilcoxon rank-sum test, Fisher exact test, CUSUM analysis	80
Chen et al. 2014 [36]	60	2	Board-certified attending general surgeons Experienced in both open and robotic surgery. In the pilot study before the present work, this surgical team had performed more than 50 robotic surgeries, including 12 PDs	Operative time, blood loss	Split group, Student’s *t*-test, chi-square test, Fisher’s exact test, Mann–Whitney *U* test, Spearman’s rank correlation coefficient, Kaplan–Meier method, log-rank test	40
Napoli et al. 2016 [22]*	70	1	-	Operative time	CUSUM	33 for operative time, 40 for readmission rate
Shyr et al. 2018 [15]	61	2	No prior experience in robotic surgery, > 500 cases of open PD	Console time	2-tailed Student’s *t*-test, Pearson’s chi-square test, Fisher’s exact test, CUSUM analysis	20
Zhang et al. 2018 [24]	100	1	Advanced open and laparoscopic skills in pancreatic surgery	Operative time, blood loss, length of hospital stay	CUSUM, Student’s *t*-test, Fisher’s exact test, chi-square test	40
Zwart et al. 2021 [25]	275	15	All surgeons had at least 5 years of experience with open pancreatic surgery. Some had experience with LPD	Operative time, blood loss, length of hospital stay, complication-related mortality	CUSUM, Pearson correlation	22

Studies marked * are conference abstracts

Boone et al. [23] performed a retrospective review of 200 patients, cared for by 4 surgeons, demonstrating a significant improvement in operative time at 80 procedures. They also subdivided their operative learning curve into three phases. Phase 1 (initial phase) was represented by the first 80 cases, phase 2 (plateau phase) is represented by case 81 to 140 and phase 3 (steady improvement) is represented by those beyond case 140. Chen et al. [36] used operative time, blood loss and morbidity to evaluate the learning curve in two surgeons and their team who had prior experience of 12 RPDs. They presented a significantly shortened mean operative time and reduced blood loss volume in the last 20 operations compared to the first 40 operations.

Napoli et al. [22] studied the learning curve in a single surgeon using the CUSUM method based on operative time and found that it dropped after the first 33 operations. Shyr et al. [15] identified the learning curve for pure RPD as 20 cases in surgeons with prior experience of RDP.

Zhang et al. [24] investigated the learning curve in a single surgeon performing his first 100 robot-assisted LPDs and demonstrated completion of the learning curve after 40 cases. Furthermore, the learning curve was subdivided into two distinct phases. Phase 1 was represented by the first 40 patients with longer operative times and phase 2 was represented by the next 60 patients which displayed a gradual improvement in operative time as the learning curve was achieved.

Zwart et al. [25] studied the learning curve in a multicentre (seven) prospective study involving 275 patients and 15 surgeons (all with at least 5 years if experience in open pancreatic surgery and some with LPD experience). The study demonstrated that the CUSUM for operative time reached an inflection point at 22 cases. The total operative time between the early and late experience group decreased significantly. The median estimated blood loss increased between the two groups but the difference was not significant.

### Quality evaluation and assessment of included studies

The majority of articles used statistical methods to demonstrate the learning curve. Four studies did not document or did not formally use any statistical methods. CUSUM analysis was used by 14 studies. The second most common type of statistical analysis was univariate split groups. This involves splitting a series of consecutive cases into two or more groups, and testing for a significant difference in the means of each group. If the mean differed for the respective outcome, the authors would infer an improvement in learning. For example, Fisher’s exact test was used by 13 studies, *χ*^**2**^ test by 10 studies, Student *t*-test by nine studies and simple ANOVA by five studies. Regression models such as linear regression and multivariable logistic regression were used by four studies. Other tools used include Mann–Whitney *U*-test, Kruskal–Wallis test and Spearman’s rank.

From the qualitative assessment (GRADEpro Guideline Development Tool) of the 30 studies, 2 were rated low, 16 moderate and 13 high (Tables 1 and 2).

### Statistical analysis

**Fig. 2:**
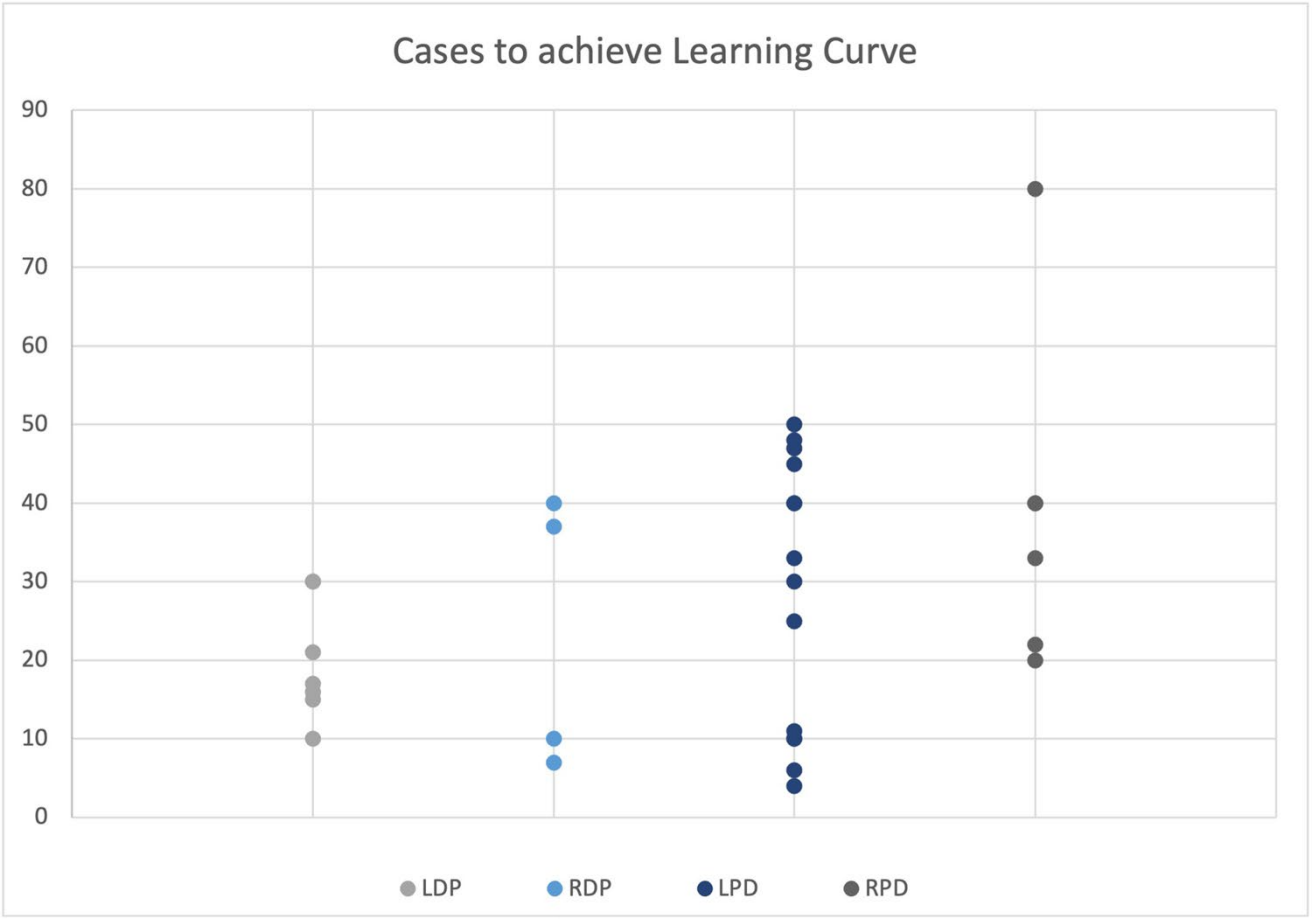
Individual learning curve plots for different modalities of DP and PD

Figure 2 illustrates the overall distribution of cases to achieve the learning curve reported in each individual study. With respect to DP, the median number of cases required in LDP and RDP was 17 (10–30) and 23.5 (7–40) respectively. In PD, the median number of cases required in LPD and RPD was 30 (4–60) and 36.5 (20–80).

**Fig. 3:**
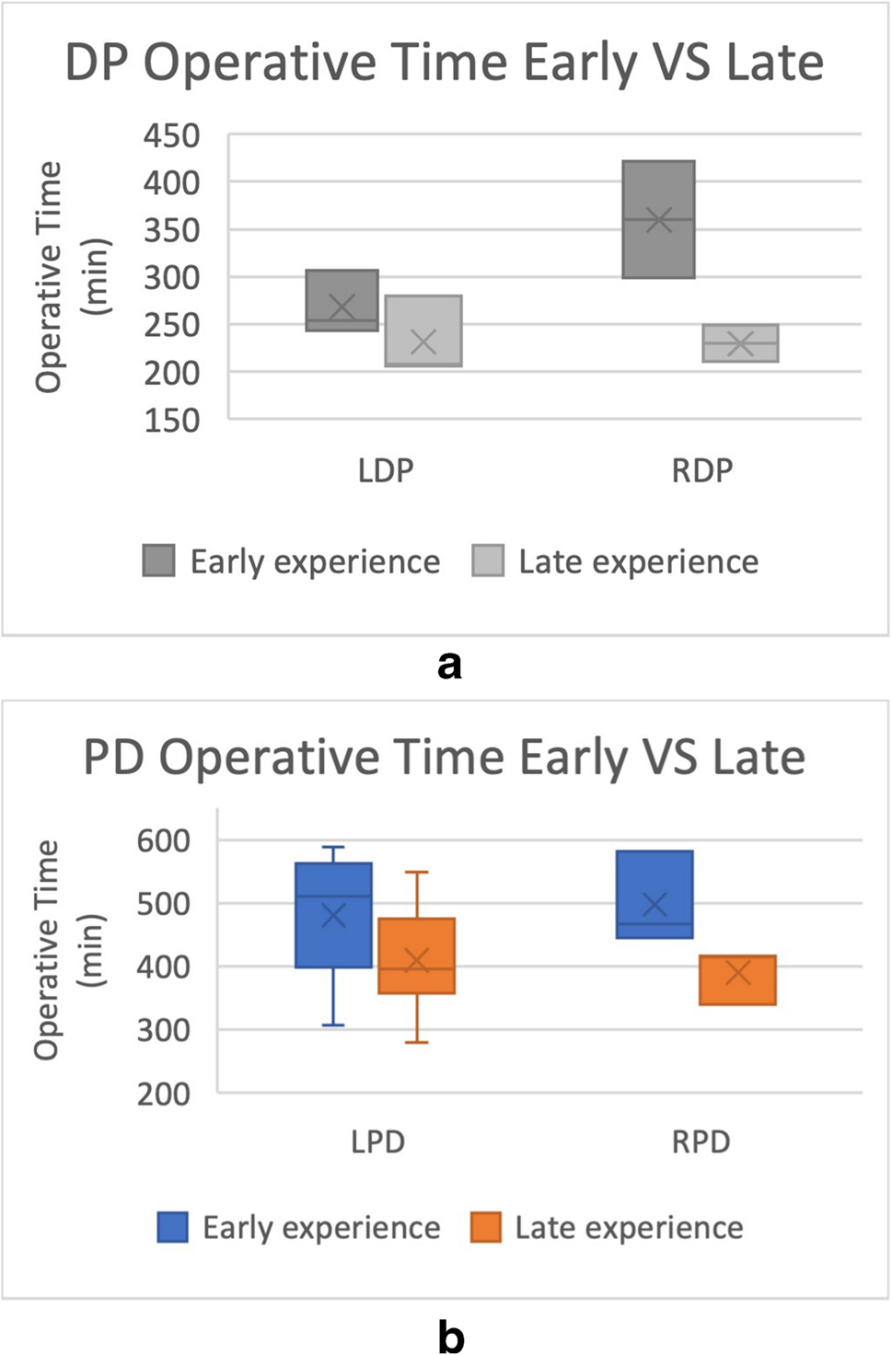
**A** Changes in operative time before and after the learning curve was achieved in LDP (*n* = 3, independent *t*-test, *p* = 0.307) and RDP (*n* = 2, independent *t*-test, *p* = 0.180). **B** Changes in operative time before and after the learning curve was achieved in LPD (*n* = 9, independent *t*-test *p* = 0.108) and RPD (*n* = 3, independent *t*-test, *p* = 0.095)

**Fig. 4:**
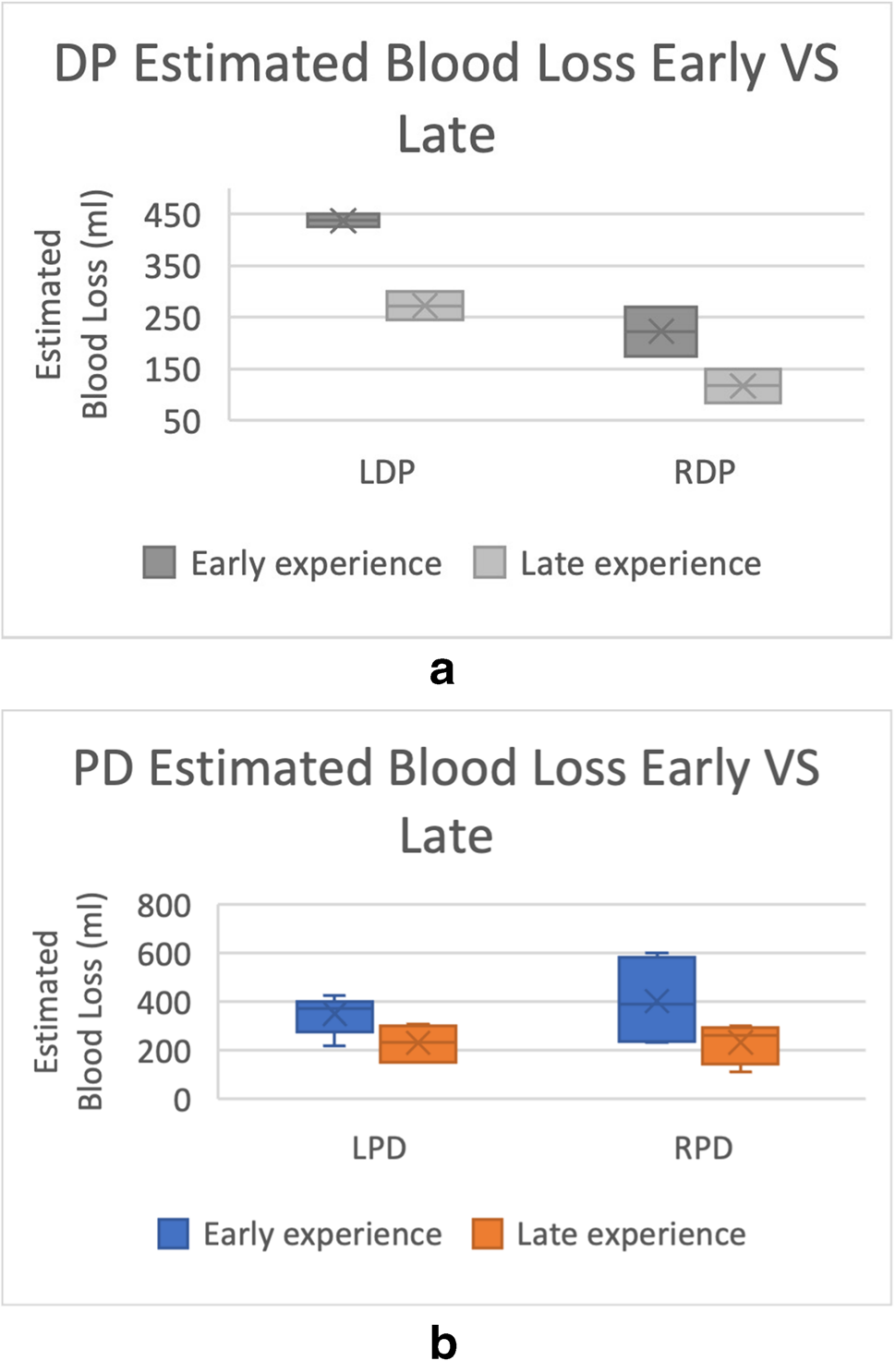
**A** Changes in estimated blood loss before and after the learning curve was achieved in LDP (*n* = 2, independent *t*-test, *p* < 0.05) and RDP (*n* = 2, independent *t*-test, *p* = 0.210). **B** Changes in estimated blood loss before and after the learning curve was achieved in LPD (*n* = 7, independent *t*-test, *p* < 0.05) and RPD (*n* = 4, independent *t*-test, *p* = 0.154). * = *p* < 0.05

On average, there is improvement in both operative parameters in (operative time and estimated blood loss) for the four modalities of operation following the achievement of the learning curve (Figs. 3 and 4). Only studies that explicitly stated measures before and after the learning curve were used for analysis.

Analyses of operative times involved a total of 17 studies (3 LDP [9–11], 2 RDP [13, 14], 9 LPD [16, 17, 19, 21, 27, 28, 30, 35, 39] and 3 RPD [23, 25, 36]) (Fig. 3). The mean operative time decreased comparing early vs late experience but were not statistically significant in all 4 groups: LDP (268.0 min vs 231.3 min, *p* = 0.307), RDP (359.85 min vs 229.65 min, *p* = 0.180), LPD (480.29 min vs 409.82 min, *p* = 0.108), RPD (497.67 min vs 390.67 min, *p* = 0.095).

Analyses of estimated blood loss involved a total of 15 studies (2 LDP [9, 10], 2 RDP [14, 15], 7 LPD [16–19, 21, 30, 35] and 4 RPD [15, 23, 25, 36]) (Fig. 4). The estimated blood loss decreased comparing early vs late experience and was statistically significant when comparing the laparoscopic study groups: LDP (437.5 ml vs 272.5 ml, *p* = 0.032), RDP (223.0 ml vs 117.5 ml, *p* = 0.210), LPD (352.0 ml vs 231.1 ml, *p* < 0.05), RPD (403.5 ml vs 234.0 ml, *p* = 0.154).

## Discussion

This systematic review shows that although many groups have attempted to quantify the learning curve in minimally invasive pancreatic surgery, these attempts vary greatly with respect to study design, reporting style and outcome measurements. This makes it rather challenging in drawing definitive conclusions.

Looking at median learning curve achievement values from Fig. 2, we can appreciate that PD has a more challenging learning period than DP (30 in LPD and 36.5 in RPD vs 17 in LDP and 23.5 in RDP). Robotic approaches in both procedures also require more cases to be performed in order to achieve the learning curve. This can be attributed to the technically demanding nature of PD and robotics. Individual surgeons might have also been new to robotic technology in some studies where not specified. Training curriculums factoring these differences will be more informed to provide more resources and opportunities for newer surgeons to become competent in such demanding procedures.

It proved challenging to collectively analyse improvements before and after the learning curve was achieved. Only 17 of the 31 studies explicitly stated the mean operative times before and after the learning curve were achieved. Likewise, only 15 of the 31 studies described the mean estimated blood loss volume before and after the learning curve was achieved in their reports. Nonetheless, from these included studies, improvements have been reported in all 4 modalities in both operative time and estimated blood loss. This shows promise in the learning curve assessment method reliably translating into tangible improvements in intraoperative outcomes.

A standardised reporting framework with explicit outcome measures across the learning curve will help facilitate substantiated cross-study comparisons that can be better applied to performance prediction.

As shown, operative time is by far the most common measure used as a surrogate marker of the learning curve. It is no doubt a convenient surrogate of proficiency; being simple to measure, continuous in nature and universally applicable.

However, beyond operative time improvements, it is important that a surgical learning curve adopts a holistic approach by factoring more patient-based outcomes. De Rooij et al. [33] further suggests that post-operative outcomes can represent a ‘proficiency curve’ whereas surgical process outcomes such as operative time, conversion rate and blood loss can represent the ‘feasibility learning curve’ [33]. They found that improvements in post-operative parameters did not correlate to their ‘feasibility learning curve’. This is supported by the fact that a few of the studies were unable to find any significant changes in operative measures where patient-based outcome improvements may have changed. This demonstrates that more consideration should be given to post-op outcomes before drawing reliable clinical conclusions.

Another factor which varied between studies was the volume of cases. As most of the studies were conducted in a single institution, whether the hospital had a high volume or low volume of surgical cases could alter the learning curve. The frequency of the performed procedure can influence the learning of the surgeon and surgeons training in a high-volume hospital will have more frequent opportunities to practise and therefore improve. Hence, the learning curve period is usually shorter in high volume than in low volume hospitals. Braga et al. [9] highlighted that having a strict selection criterion can also shorten the learning curve by avoiding a high number of unsuccessful procedures. Speicher et al. [30] also had selection criteria which involved selecting less challenging cases for the laparoscopic approach which could bias the results. Therefore, results may not necessarily extend to all institutions.

Another limitation is the varying experience of surgeons in each study. Barrie et al. [8] acknowledged that they produced a relatively short learning curve as their operating surgeons already had extensive prior experience in complex laparoscopic procedures. Therefore, the results may not be applicable to every surgeon, especially since trainees attempting to identify their own position on the learning curve are likely inexperienced themselves. It is possible that learning curves are based on innate ability, training level and prior laparoscopic experience and will remain difficult to control in studies. Despite the prior experience of the learner being a significant confounding factor, many studies did not describe the background experience of the surgeon that can allow for qualifying outcomes. Furthermore, skills are translatable as a surgeon with robotic gastro-intestinal experience without pancreatic surgical experience may be at an advantage over another surgeon without any robotic experience. This would be a confounding factor if a study does not account for this in their analysis. We stress the need for future studies to further qualify the experience of surgeons involved in order to improve the generalisability of their results.

It is also important for us to contextualise the study as to when it was conducted as this can influence when the learning curve was reached. For example, Kim et al. [39] study began in 2007 and one can argue that it was more challenging to reach the learning for LPD back then compared to 2021. Recent studies may also involve surgeons and centres more experienced with robotic procedures in general which can contribute to the learning curve in RPD and RDP being achieved earlier.

CUSUM analysis remains a popular method for monitoring surgical success; it is effective at identifying a decline or improvements in performance; however, it performs less well than univariate split groups. Also, there may be room for bias as the acceptable failure rate for the particular outcome in question is investigator dependent. In the studies which used split group analysis, papers often gave no rationale for the method used to divide up the group rather arbitrarily which could introduce bias as well. Furthermore, with this method, it is difficult to define the actual learning ‘curve’, making it less useful for surgeons who wish to identify their position on the curve.

Another group [40] studying the learning curve in minimally invasive paediatric surgery has proposed a framework for reporting the learning curve. This framework involves three domains: measuring, presenting and interpreting. They postulate that at least one outcome measure should be universal, the learning curve should be ‘presented’ graphically with adequate statistical analysis and the impact of said learning curve should be ‘interpreted’ with regard to its relevance in clinical practice. Although in this method where study designs may still vary, standardised reporting methods could greatly increase the usability of learning curve data. Following this proforma could be a step towards standardisation of learning curve studies in all surgical specialities.

The information pertaining to the learning curve in minimally invasive pancreatic surgery can not only be used as a guide for training surgeons to gauge their own performance but can also be used in the decision-making of adopting newer innovative techniques especially in lower volume hospitals. Cost-effectiveness is a central and unavoidable metric which should be considered in the adoption of new procedures and the learning curve plays a major role in determining cost-effectiveness. Previous studies have shown that shortening the learning curve is critical in managing the costs of laparoscopic surgery.

Lastly, this review focused on distal pancreatectomies and pancreaticoduodenectomies due to its popularity. It would be interesting and useful to see more data on the learning curves of less common procedures such as minimally invasive total pancreatectomy, enucleation and middle pancreatectomy and account for any differences.

## Conclusion

At present, there are multiple studies describing the learning curve in minimally invasive pancreatic surgery. The median learning curve achievement point for distal pancreatectomy was reported to be 17 (10–30) cases for laparoscopic and 23.5 (7–40) cases for robotic. The median learning curve achievement point for pancreaticoduodenectomy was reported to be 30 (4–60) cases for laparoscopic and 36.5 (20–80) cases for robotic. Most learning curve studies are based upon surgical process outcomes. This review has highlighted the need for a standardised method for reporting learning curves to compensate for the complexities of confounding factors between operations.
